# Submembrane ATP and Ca^2+^ kinetics in α-cells: unexpected signaling for glucagon secretion

**DOI:** 10.1096/fj.14-265918

**Published:** 2015-04-24

**Authors:** Jia Li, Qian Yu, Parvin Ahooghalandari, Fiona M. Gribble, Frank Reimann, Anders Tengholm, Erik Gylfe

**Affiliations:** *Department of Medical Cell Biology, Uppsala University, Uppsala, Sweden; and ^†^Cambridge Institute for Medical Research, Addenbrooke's Hospital, Cambridge, United Kingdom

**Keywords:** oscillations, islet of Langerhans, signal transduction, paracrine

## Abstract

Cytoplasmic ATP and Ca^2+^ are implicated in current models of glucose’s control of glucagon and insulin secretion from pancreatic α- and β-cells, respectively, but little is known about ATP and its relation to Ca^2+^ in α-cells. We therefore expressed the fluorescent ATP biosensor Perceval in mouse pancreatic islets and loaded them with a Ca^2+^ indicator. With total internal reflection fluorescence microscopy, we recorded subplasma membrane concentrations of Ca^2+^ and ATP ([Ca^2+^]_pm_; [ATP]_pm_) in superficial α- and β-cells of intact islets and related signaling to glucagon and insulin secretion by immunoassay. Consistent with ATP’s controlling glucagon and insulin secretion during hypo- and hyperglycemia, respectively, the dose-response relationship for glucose-induced [ATP]_pm_ generation was left shifted in α-cells compared to β-cells. Both cell types showed [Ca^2+^]_pm_ and [ATP]_pm_ oscillations in opposite phase, probably reflecting energy-consuming Ca^2+^ transport. Although pulsatile insulin and glucagon release are in opposite phase, [Ca^2+^]_pm_ synchronized in the same phase between α- and β-cells. This paradox can be explained by the overriding of Ca^2+^ stimulation by paracrine inhibition, because somatostatin receptor blockade potently stimulated glucagon release with little effect on Ca^2+^. The data indicate that an α-cell-intrinsic mechanism controls glucagon in hypoglycemia and that paracrine factors shape pulsatile secretion in hyperglycemia.—Li, J., Yu, Q., Ahooghalandari, P., Gribble, F. M., Reimann, F., Tengholm, A., Gylfe, E. Submembrane ATP and Ca^2+^ kinetics in α-cells: unexpected signaling for glucagon secretion.

Insulin and glucagon have central roles in maintaining normal glycemia by lowering and increasing blood glucose, respectively. The circulating concentrations of both hormones oscillate (1, 2), which promotes hormone action by keeping the receptors up-regulated (3). Consistent with their opposing effects, the oscillations of circulating insulin and glucagon are synchronized in opposite phase (1, 2), as is the underlying pulsatile release of insulin and glucagon from isolated human (4) or mouse (5) pancreatic islets. In type 2 diabetes, the regular insulin oscillations deteriorate (6, 7), and prediabetes is associated with loss of the characteristic phase relationship between the insulin and glucagon oscillations (2). These changes may contribute to β-cell exhaustion and diabetes, because more insulin is needed after down-regulation of its receptors, and glucose storage/production by the liver is perturbed when insulin and glucagon no longer vary in opposite phase.

There is consensus about the central mechanisms underlying glucose regulation of insulin secretion from β-cells, with rapid uptake and metabolism of the sugar, generation of ATP, and closure of ATP-sensitive K^+^ (K_ATP_) channels resulting in depolarization with influx of secretion-triggering Ca^2+^ through voltage-dependent channels (8). In contrast, the processes by which glucagon release from α-cells is stimulated in response to hypoglycemia and is inhibited when normal glycemia is reestablished continue to puzzle the scientific community. Glucose may act indirectly *via* autonomic (9, 10) and paracrine (11–15) mechanisms, but there is also strong evidence of direct glucose sensing by the α-cells (16–20). ATP is also a key player in different models of glucose-regulated glucagon secretion from the α-cell, but its role varies considerably. Glucose-generated ATP has thus been thought to mediate reduction of voltage-dependent Ca^2+^ influx and exocytosis in α-cells (21) by α-cell hyperpolarization induced by providing energy to the electrogenic Na^+^/K^+^ pump (16) or by shutting off a depolarizing store-operated current after energizing sarco(endo)plasmic Ca^2+^-ATPase (18, 20). It has also been suggested that glucose-induced elevation of the ATP/ADP ratio, as in β-cells, closes K_ATP_ channels to depolarize the α-cells, which paradoxically inhibits voltage-dependent Ca^2+^ influx and glucagon release (17, 19). A fourth alternative is that the glucose-induced elevation of ATP is associated with a reduction of AMP-activated protein kinase activity, which inhibits glucagon release by a mechanism that may be partly Ca^2+^ independent (22). Although all these models involve glucose-induced generation of ATP, relatively little is know about ATP kinetics in the α-cell. Measurements on purified rat islet cell populations confirmed that an increase in glucose concentration raises ATP and the ATP/ADP ratio in β-cells, but there are no changes in the nucleotides in the α-cells, which already have a relatively high ATP/ADP ratio at low glucose concentrations (23). In later studies of mouse islets with luciferase-expressing α-cells, there were modest elevations of ATP in response to 15–20 mM glucose (11, 14) concentrations, much higher than the 7–8 mM that maximally inhibits secretion (20, 24). Recently, changes in glucose concentration of between 1 and 6 mM were found to induce reversible responses of the ATP-binding fluorescent probe Perceval in red fluorescent protein (RFP)-expressing α-cells of transgenic GLU-RFP mice (mice expressing RFP under proglucagon promoter control) (25).

In the present study, we used Perceval (26) and total internal reflection fluorescence (TIRF) microscopy to monitor the ATP concentration in the subplasma membrane space ([ATP]_pm_) of peripheral cells in mouse pancreatic islets. Supporting a role of α-cell ATP in glucagon-mediated glucose counterregulation, [ATP]_pm_ in α-cells was relatively more sensitive than that in β-cells, in response to the low glucose concentrations that characterize hypoglycemia. Both α- and β-cells showed oscillations of [ATP]_pm_ that were in opposite phase to those of the Ca^2+^ concentration in the subplasma membrane space ([Ca^2+^]_pm_) indicating energy-dependent Ca^2+^ transport. Although 20 mM glucose induces a pulsatile release of insulin and glucagon in opposite phase (4, 5), this glucose concentration tended to synchronize the [Ca^2+^]_pm_ oscillations in α- and β-cells in phase. Because oscillatory Ca^2+^ peaks drive the insulin pulses (27, 28), those of glucagon must occur during Ca^2+^ nadirs. This paradox is attributable to Ca^2+^-independent paracrine inhibition by somatostatin, because a somatostatin receptor (SSTR) type 2 antagonist potently stimulated glucagon release with little effect on α-cell [Ca^2+^]_pm_.

## MATERIALS AND METHODS

### Materials and experimental medium

The primary polyclonal rabbit anti-insulin antibody was from Abcam (Cambridge, United Kingdom), and the primary polyclonal rabbit anti-glucagon antibody was from Dako (Carpinteria, CA, USA). The secondary antibody Alexa Flour 488 goat anti-rabbit IgG was from Life Technologies (Rockville, MD, USA). Poly-l-lysine, diazoxide, glutamic acid, and HEPES were from Sigma-Aldrich (St. Louis, MO, USA). Fetal bovine serum (FBS) was from Life Technologies-Gibco (Grand Island, NY, USA). The insulin and SSTR-2 antagonists S961 and PRL2903 were kind gifts from Novo Nordisk, Bagsværd, Denmark, and Dr. D. H. Coy (Tulane University, New Orleans, LA, USA), respectively. Adenovirus encoding the fluorescent ATP biosensor construct Perceval (26) was used according to a published method (29). Superfusion and batch incubation of islets were made with experimental medium containing 138 mM NaCl, 4.8 mM KCl, 1.2 mM MgCl_2_, 1.3 or 2.6 (hormone release) mM CaCl_2_, 3 mM glucose, 0.5 mg/ml BSA, and 25 mM HEPES with pH adjusted to 7.4 with NaOH.

### Animals, islet isolation, cell culture, and virus infection

All animal experimental procedures were approved by the local ethics committee for use of laboratory animals in Uppsala, Sweden. Transgenic mice with homozygous expression of RFP in α-cells activated by proglucagon promoter-driven Cre-mediated excision of a stop codon (GLU-RFP mice) were produced in Cambridge (30), followed by backcrossing for 8 generations into the C57Bl/6J strain and continued breeding in Uppsala. Normal C57Bl/6J mice were obtained from Taconic (Ry, Denmark). Islets of Langerhans were isolated by collagenase digestion of the pancreas (20) from 4- to 12-mo-old mice. After isolation, the islets were cultured for 18–24 h in RPMI 1640 medium containing 5.5 mM glucose, 10% FBS, 100 units/ml penicillin, and 100 μg/ml streptomycin, at 37°C in an atmosphere of 5% CO_2_ in humidified air.

For ATP measurements, the islets were infected with the Perceval adenovirus at concentrations of 1–2 × 10^6^ plaque-forming units/islet or 20 plaque-forming units/cell for 1–2 h. During infection, the concentration of serum was reduced to 2%. The islets were subsequently washed 3 times with normal RPMI 1640 medium and cultured for at least 18 h before use.

### Measurements of [Ca^2+^]_pm_

Islets from transgenic GLU-RFP mice were incubated for 20–30 min with 1.2 μM acetoxymethyl ester of the Ca^2+^ indicator Fluo-4 (Life Technologies). The islets were allowed to attach onto poly-l-lysine-coated coverslips for 5–10 min. Coverslips with Fluo-4-loaded islets were used as exchangeable bottoms of an open 50 µl chamber that was superfused with experimental medium at a rate of 0.12–0.2 ml/min at 37°C. [Ca^2+^]_pm_ was then measured with TIRF microscopy (31).

### Parallel measurements of [ATP]_pm_ and [Ca^2+^]_pm_

Because of interference between Perceval and RFP, ATP could not be measured reliably in α-cells from transgenic GLU-RFP mice (see below). Most ATP measurements were therefore made on islets from normal mice. Before experiments Perceval-infected islets were preincubated for 60 min in experimental medium containing 5 μM of the acetoxymethyl ester of the Ca^2+^ indicator Fura Red (Life Technologies). The same experimental conditions were used as when measuring [Ca^2+^]_pm_ alone. Perceval and Fura Red fluorescence were excited at 491 nm by a diode-pumped solid-state laser (Cobolt AB, Stockholm, Sweden), and fluorescence was measured with 530/50 nm (Perceval) and 620 nm long-pass (Fura Red and RFP) filters (Semrock, Rochester, NY, USA). Image pairs were acquired every 5 s, as described above.

### Immunostaining

Immunostaining was performed at room temperature, unless otherwise stated, with PBS. Isolated islets were thoroughly rinsed and then fixed with 4% paraformaldehyde for 10 min, followed by cell permeabilization by exposure to 0.2% Triton X-100 on ice for 10 min. Nonspecific antigens were blocked by 30 min exposure to 5% FBS (blocking buffer). The islets were then incubated for 2 h with rabbit polyclonal anti-insulin or -glucagon diluted 1:100 in blocking buffer followed by 3 washes. Subsequently, the islets were incubated in darkness for 1 h with Alexa Fluor 488 goat anti-rabbit IgG diluted 1:200 in blocking buffer. The islets were then washed 4 times for 5 min in 0.1% Tween-20 to reduce background, followed by 3 additional washings in PBS.

### Confocal microscopy

A previously described confocal microscope setup (29) was used to image immunostained islets with excitation/emission at 488/527 nm for Alexa Fluor 488 and 561 and/or >645 nm for RFP.

### Measurements of hormone release

Batches of 8–10 size-matched islets were preincubated at 37°C for 30 min in experimental medium containing 3 mM glucose followed by incubation for 40 min in 500 μl medium containing 3 or 20 mM glucose and hormone receptor antagonists, as indicated in Fig. 6. The incubation medium was then collected, and the islets were briefly sonicated in acid ethanol. Samples from the medium and islets were appropriately diluted and taken for duplicate assays of glucagon and insulin. Glucagon was measured with an ELISA kit from Mercodia AB (Uppsala, Sweden), according to instructions, and insulin was determined with a mouse/rat insulin immunoassay kit from Mesoscale Discovery (Rockville, MD, USA) with custom-optimized protocol. Secretion was then expressed as a percentage of total hormone content.

### Data and statistical analysis

Image analysis was conducted with MetaFluor (Molecular Devices Corp, Sunnyvale, CA, USA) or FIJI (32) software. Igor Pro software (Wavemetrics, Lake Oswego, OR, USA) was used to correct for photobleaching and extrusion of the indicators Fluo-4 and Fura Red, assuming exponential fluorescence decay. Normalization to conditions when [Ca^2+^]_pm_ and [ATP]_pm_ were low was used to compensate for variations in cellular content of the different fluorescent indicators. To facilitate a [Ca^2+^]_pm_ comparison between α- and β-cells, the Fluo-4 fluorescence was expressed as changes in relation to fluorescence after background subtraction observed in Ca^2+^-deficient medium containing EGTA at the end of the experiments (*F*/*F*_Ca0_). The Fura Red fluorescence, which decayed more rapidly, was instead expressed in relation to the initial fluorescence in the presence of 3 mM glucose when [Ca^2+^]_pm_ was low (*F*/*F*_0_) in the β-cells. However, in α-cells with considerable [Ca^2+^]_pm_ activity at different glucose concentrations, Fura Red fluorescence was instead expressed in relation to fluorescence at the lowest [Ca^2+^]_pm_ values during each recording (*F*/*F*_CaLow_). Since Fura Red fluorescence decreases upon Ca^2+^ binding, the traces were inverted (mirrored) to show increases in [Ca^2+^]_pm_ as positive deflections. Perceval and RFP fluorescence data were always expressed as changes in relation to initial fluorescence after subtraction of background (*F*/*F*_0_). The relationships between [Ca^2+^]_pm_ and [ATP]_pm_ oscillations in α- and β-cells were analyzed with asymmetric sliding-window cross-correlation (33) in MATLAB (MathWorks Inc., Natick, MA, USA). Igor Pro (Wavemetrics) and Illustrator (Adobe Systems, San Jose, CA, USA) were used for the illustrations. Data are presented as means ± sem. Statistical comparisons between 2 groups were assessed with paired *t* tests and multiple comparisons with ANOVA followed by *post hoc* paired *t* tests with the Holm-Bonferroni sequential correction.

## RESULTS

### Identification of α- and β-cells

**Figure 1.**
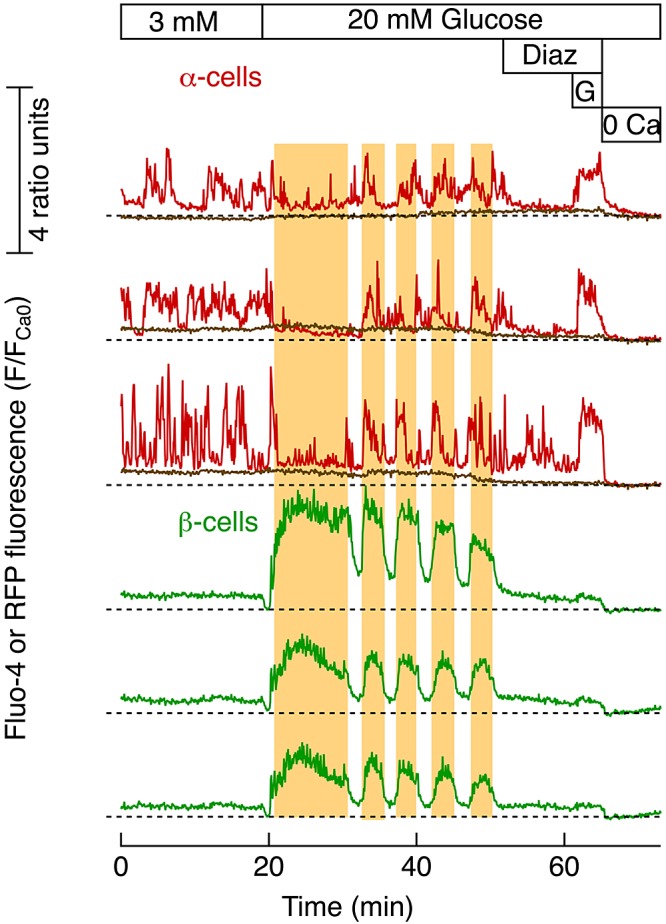
[Ca^2+^]_pm_ responses to glucose and glutamate can be used to identify α- and β-cells. TIRF microscopy was used to record [Ca^2+^]_pm_ in individual cells within a pancreatic GLU-RFP mouse pancreatic islet loaded with the indicator Fluo-4. Fluorescence is normalized as the *F*/*F*_Ca0_ ratio, where *F*_Ca0_ is the fluorescence in the absence of extracellular Ca^2+^ at the end of the experiment. Glucose elevation from 3 to 20 mM temporarily inhibited oscillatory [Ca^2+^]_pm_ signaling in 3 α-cells (red) without affecting α-cell RFP fluorescence (brown) and induced synchronized [Ca^2+^]_pm_ oscillations in 3 β-cells (green) within the same pancreatic islet. At the end of the experiment, 250 μM of the hyperpolarizing K_ATP_ channel activator diazoxide (Diaz) was added, followed by 1 mM glutamate (G) and omission of extracellular Ca^2+^, which was combined with the addition of 1 mM EGTA (0 Ca). Vertical scale bar: 4 *F*/*F*_Ca0_ ratio units; dotted lines: unity ratio for the trace above. The vertical yellow background areas are aligned to glucose-induced peaks of the [Ca^2+^]_pm_ oscillations in the β-cell.

To facilitate cell identification, islets were isolated from GLU-RFP mice with RFP-expressing α-cells. Confocal imaging of immunostained islets showed that nearly all of the RFP-positive and -negative cells were α- and β-cells, respectively (Supplemental Fig. S1). However, unexpected problems became apparent after infection with the ATP sensor Perceval, which interacted with RFP to produce erratic responses affecting both fluorophores in the α-cells (Supplemental Fig. S2). Because the RFP did not interact with the Ca^2+^ indicator Fluo-4, we instead used the transgenic mouse islets and TIRF microscopy to establish functional criteria for discriminating between α- and β-cells, based on characteristic [Ca^2+^]_pm_ responses. Most RFP-negative cells had low, stable [Ca^2+^]_pm_ levels in 3 mM glucose, and introduction of 20 mM glucose caused a β-cell-characteristic (34) initial decrease in Ca^2+^, followed by prompt and prolonged elevation and pronounced, regular, slow oscillations that were almost perfectly synchronized between the different β-cells (**Fig. 1**). The RFP-positive α-cells with a smaller footprint area than the RFP-negative cells showed [Ca^2+^]_pm_ activity in 3 mM glucose with nonsynchronized irregular peaks. Elevation to 20 mM, which did not affect RFP fluorescence, often resulted in inhibition of the [Ca^2+^]_pm_ activity parallel to the prolonged [Ca^2+^]_pm_ elevation in the β-cells. The subsequently recurring [Ca^2+^]_pm_ oscillations did not exhibit obvious synchronization. Addition of 250 μM diazoxide, which hyperpolarizes by activating K_ATP_ channels, reduced [Ca^2+^]_pm_ to basal levels in all β- and many α-cells. Glutamate activates ionotropic receptors in α- but not in β-cells (35), and subsequent addition of 1 mM of this amino acid depolarized most RFP-positive α-cells sufficiently to induce rapid [Ca^2+^]_pm_ elevation, but had a very modest effect on the β-cells. This glutamate response was observed in 135 of 161 RFP-positive cells (84%) with [Ca^2+^]_pm_ activity at 3 mM glucose in islets from 15 mice. Cell footprint area, the [Ca^2+^]_pm_ patterns in 3 and 20 mM glucose, and glutamate responsiveness were therefore always used to discriminate between α- and β-cells.

### The ATP response to glucose is left shifted and less pronounced in α- than in β- cells

**Figure 2.**
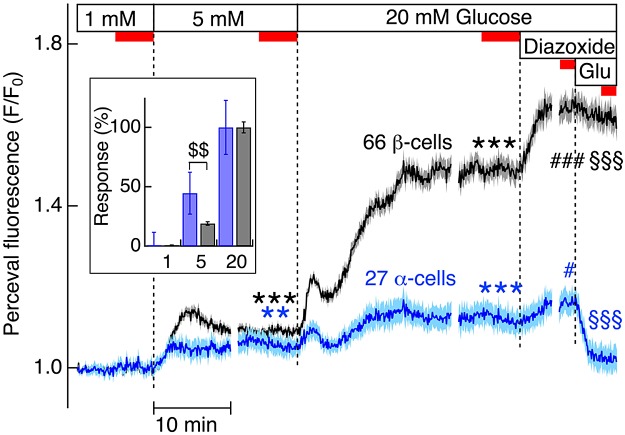
α-Cell [ATP]_pm_ increases less but is relatively more sensitive to low glucose concentrations than that in β-cells. TIRF microscopy recorded Fura Red (not shown) and Perceval fluorescence in individual cells within 13 pancreatic islets from 8 normal mice. Average [ATP]_pm_ data (dark blue: α-cells; black: β-cells) ± sem (light blue: α-cells; gray: β-cells) for 27 α- and 66 β-cells. The islets were initially exposed to 1 mM glucose, and the concentration was then increased to 5 and 20 mM, as indicated. At the end of the experiment, 250 μM diazoxide was added followed by 1 mM glutamate (Glu). Because of timing differences between experiments, the traces are broken and aligned to the time points for media changes (dotted lines). Oscillations were essentially cancelled out when data were averaged from several experiments with differences in timing. The effects of increasing the glucose concentration and adding diazoxide and glutamate were evaluated by comparing average data during the periods indicated (red bars). Perceval fluorescence (F) is normalized as the *F*/*F*_0_ ratio, where *F*_0_ is the initial fluorescence. Inset: steady-state [ATP]_pm_ at 5 mM glucose in α- (blue) and β- (gray) cells after normalization to the levels at 1 and 20 mM glucose (0 and 100%, respectively). ^$$^*P* < 0.005, normalized response to 5 mM glucose of α- *vs.* β-cells. ***P < 0.001, **P < 0.005, effect of 5 or 20 mM *vs.* mM glucose. ^###^*P* < 0.001, ^#^*P* < 0.05, effect of diazoxide *vs*. 20 mM glucose. ^§§§^*P* < 0.001, effect of glutamate *vs.* diazoxide. Blue: α-cells; black: β-cells.

Subsequent studies of [ATP]_pm_ kinetics were made with Perceval-infected islets from regular C57Bl/6J mice. To avoid spectral overlap with Perceval fluorescence, we used the Ca^2+^ indicator Fura Red for the cell identification with [Ca^2+^]_pm_ recordings. Even though Fura Red is inferior to Fluo-4, with lesser changes in fluorescence intensity, higher Ca^2+^ affinity, and more rapid photobleaching, it served rather well together with Perceval, and there was no interference between the probes (see below). Considering the implication of ATP in glucose-regulated glucagon and insulin release, we next compared [ATP]_pm_ in the 2 cell types at glucose concentrations covering the glucose control range for both hormones (**Fig. 2**). Increase in glucose from 1 to 5 mM, which induces pronounced inhibition of glucagon release but fails to stimulate insulin secretion from mouse islets (20, 24), caused a similarly rapid elevation of Perceval fluorescence in α- and β-cells (Fig. 2). Whereas the modest α-cell response was close to maximal after 2–3 min, Perceval fluorescence continued to increase for 5 min in the β-cells and then declined to a level corresponding to 9% above the initial baseline, as compared to 5% in the α-cells (*P* < 0.001). Further glucose elevation to 20 mM to stimulate insulin release caused a more pronounced increase in Perceval fluorescence in β- than in α-cells (49% *vs.* 12% above baseline; *P* < 0.001; Fig. 2). However, the relative increase in Perceval fluorescence in 5 mM glucose was greater in α- than in β-cells (45% *vs.* 19% of the 20 mM glucose response in each cell type, respectively; Fig. 2, inset). These data indicate that ATP increased considerably more in β- than in α-cells and that the α-cells showed left-shifted glucose concentration dependence. Subsequent hyperpolarization with diazoxide, which lowered [Ca^2+^]_pm_ in both α- and β-cells, caused an increase in Perceval fluorescence in both cell types, and under those conditions, glutamate induced a rapid decrease in Perceval fluorescence only in the α-cells (Fig. 2), likely reflecting the concomitant [Ca^2+^]_pm_ elevation. A much slower and gradual, but statistically significant, reduction of Perceval fluorescence in the β-cells can be attributed to the time-dependent reduction of metabolism when Ca^2+^ entry was blocked by diazoxide (29).

### Glucose induces synchronized ATP oscillations in α- and β-cells with Ca^2+^ oscillating in opposite phase

We next investigated the relationships between [ATP]_pm_ and [Ca^2+^]_pm_ in α- and β-cells by correlating Fura Red and Perceval fluorescence kinetics in the different cell types within individual islets. Supplemental Fig. S3 shows average traces from 11 β-cells in the same islet, with an oscillatory [ATP]_pm_ response to 20 mM glucose (4.5 min periodicity) similar to that in the GLU-RFP mouse β-cells (Supplemental Fig. S2). The simultaneous [ATP]_pm_ and [Ca^2+^]_pm_ recordings reinforced our previous observations (29) that the oscillations are essentially antiparallel. This result is apparent from the 2-dimensional cross-correlogram, with strong negative correlation between the [ATP]_pm_ and [Ca^2+^]_pm_ oscillations when the traces were not time shifted and strong positive correlation when the traces were shifted by half an oscillatory period. The first elevation of [ATP]_pm_ coincided with an initial [Ca^2+^]_pm_ reduction, and the subsequent first increase in [Ca^2+^]_pm_ caused a temporary interruption of the [ATP]_pm_ increase. During established oscillations, the increase in [Ca^2+^]_pm_ slightly preceded the lowering of [ATP]_pm_. Glutamate had no apparent effect on the glucose-induced [Ca^2+^]_pm_ or [ATP]_pm_ oscillations (Supplemental Fig. S3).

**Figure 3.**
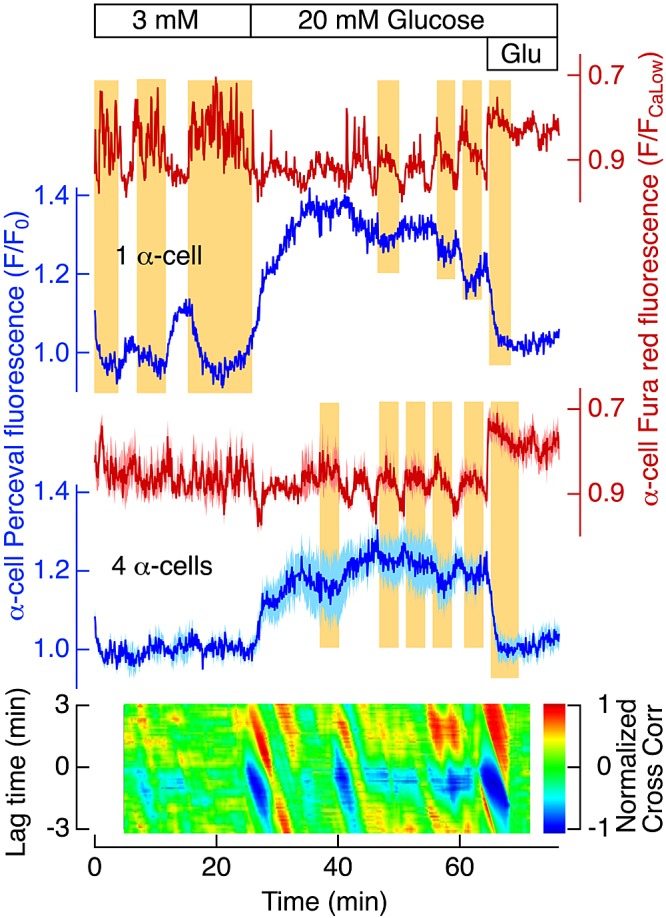
Glucose elevation raises α-cell [ATP]_pm_ and induces oscillations in opposite phase to those of [Ca^2+^]_pm_. TIRF microscopy recorded Fura Red and Perceval fluorescence in individual cells within a pancreatic islet from a normal mouse. Perceval fluorescence (F) is normalized as the *F*/*F*_0_ ratio, where *F*_0_ is the initial fluorescence and Fura Red fluorescence is normalized to that corresponding to the lowest [Ca^2+^]_pm_ values (*F*/*F*_CaLow_). The Fura Red scale is inverted to show increases of [Ca^2+^]_pm_ as positive deflections. Top traces: [Ca^2+^]_pm_ (red) and [ATP]_pm_ (blue) data for a single α-cell; bottom traces: average data for 4 α-cells within an islet (red: [Ca^2+^]_pm_; dark blue: [ATP]_pm_) ± sem (pink: [Ca^2+^]_pm_; light blue: [ATP]_pm_). The islet was initially exposed to 3 mM glucose, and the concentration was then increased to 20 mM, as indicated. At the end of the experiment, 1 mM glutamate (Glu) was added. The vertical yellow background areas are aligned to increases of the [Ca^2+^]_pm_ that correspond to decreases in [ATP]_pm_. The correlation of the average data was calculated from consecutive pairs of data segments of 4 min duration and shifted 5 s in time in relation to the previous segment. A 2-dimensional cross-correlogram (major colored area) was constructed from consecutive 1-dimensional cross-correlations with time on the *x*-axis and the lag time of the correlation on the *y*-axis and the normalized cross-correlation amplitude coded in color (vertical color bar).

**Figure 3** shows similar antiparallel relationships between [Ca^2+^]_pm_ and [ATP]_pm_ oscillations in a single α-cell and after averaging data from 4 α-cells within the same islet as the β-cells in Supplemental Fig. S3. In 3 mM glucose, the antiparallel relationship was obvious only when we analyzed single α-cells; both [Ca^2+^]_pm_ and [ATP]_pm_ oscillations tended to cancel out when data were averaged from several cells. However, in 20 mM glucose, the antiparallel [Ca^2+^]_pm_ and [ATP]_pm_ oscillations became sufficiently synchronized to be apparent, also after data were averaged from several α-cells, with identical periodicity but much smaller amplitudes, as compared to that of β-cells. The cross-correlogram showed that the phase relationship between the [Ca^2+^]_pm_ and [ATP]_pm_ oscillations in α-cells was very similar to that in β-cells. However, glutamate induced a pronounced increase in [Ca^2+^]_pm_ and a lowering of [ATP]_pm_ levels only in the α-cells (Fig. 3).

**Figure 4.**
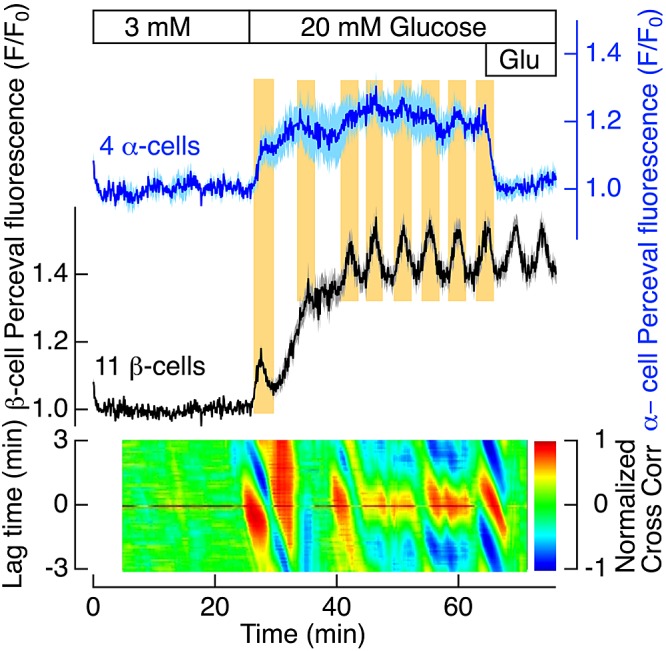
Glucose-induced [ATP]_pm_ oscillations are synchronized between α- and β-cells. TIRF microscopy recorded Perceval fluorescence in individual cells within a pancreatic islet from a normal mouse. Perceval fluorescence (F) is normalized as the *F*/*F*_0_ ratio, where *F*_0_ is the initial fluorescence. Shown are the average [ATP]_pm_ data (dark blue: α-cells; black: β-cells) ± sem (light blue: α-cells; gray: β-cells) for 4 α- and 11 β-cells within the same islet (as also shown in Supplemental Figs. S3, S4). The islet was initially exposed to 3 mM glucose, and the concentration was then increased to 20 mM, as indicated. At the end of the experiment 1, mM glutamate (Glu) was added. The vertical yellow background areas are aligned to coinciding increases in [ATP]_pm_ in the α- and β-cells. The 2-dimensional cross-correlogram (major colored area) was constructed as described in Fig. 3.

When we compared the [ATP]_pm_ kinetics in the 4 α- and 11 β-cells within the same islet, it became evident that oscillations induced by 20 mM glucose are synchronized between the 2 cell types, with the highest positive correlation when the traces were not time shifted (**Fig. 4**). It follows from the antiparallel relationship between [ATP]_pm_ and [Ca^2+^]_pm_ oscillations in either α- or β- cells and parallel [ATP]_pm_ oscillations in the 2 cell types that the [Ca^2+^]_pm_ oscillations also must be synchronized in the same phase between α- and β-cells, which is evident in Supplemental Fig. S4. The data shown in Figs. 3 and 4 and Supplemental Figs S3 and S4 were obtained from the same pancreatic islet, but similar relationships between [ATP]_pm_ and [Ca^2+^]_pm_ oscillations in α- and β-cells were observed in another 7 islets from 6 different mice.

**Figure 5.**
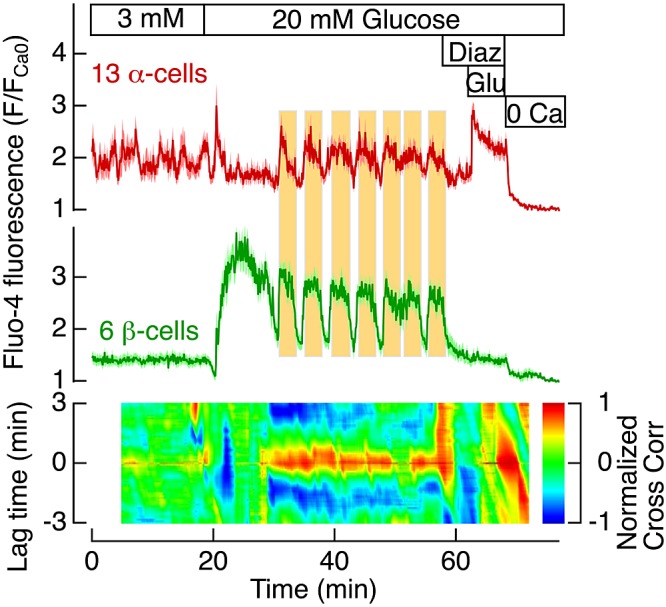
[Ca^2+^]_pm_ oscillations in α- and β-cells become synchronized at high glucose concentrations. Average [Ca^2+^]_pm_ data (dark red: α-cells; dark green: β-cells) ± sem (pink: α-cells; light green: β-cells) are shown for all 13 α- and 6 β-cells within a single GLU-RFP mouse islet. Fluorescence is normalized as the *F*/*F*_Ca0_ ratio where *F*_Ca0_ is the fluorescence in the absence of extracellular Ca^2+^ at the end of the experiment. The vertical yellow background areas are aligned to glucose-induced peaks of the [Ca^2+^]_pm_ oscillations in the β-cell. Correlation was calculated from consecutive pairs of data segments of 4 min duration and shifted 5 s in relation to the previous segment. The 2-dimensional cross-correlogram (major colored area) was constructed as described in the legend to Fig. 3.

Insulin secretion from human (4) and mouse (5) pancreatic islets exposed to 20 mM glucose is pulsatile, and studies of mouse islets and insulinoma cells have demonstrated that secretory pulses are driven by synchronous oscillations of the cytoplasmic concentrations of Ca^2+^ and cAMP (28, 31). In addition, glucagon secretion is pulsatile at 20 mM glucose, and the pulses are in opposite phase to those of insulin (4, 5). Because Ca^2+^ is supposed to be an equally important trigger of glucagon release, the synchronized [Ca^2+^]_pm_ oscillations between α- and β-cells is utterly surprising. To challenge this unexpected finding, we performed additional [Ca^2+^]_pm_ recordings with the superior Fluo-4 indicator in the transgenic GLU-RFP mice. **Figure 5** shows data from 13 α- and 6 β-cells in the same islet, providing additional evidence that glucose induces synchronized [Ca^2+^]_pm_ oscillations in the 2 cell types. Similar synchronization was observed in another 36 experiments with Fluo-4-loaded islets from 21 different GLU-RFP mice.

### An SSTR-2 antagonist potently stimulates glucagon and insulin secretion but has only modest effects on [Ca^2+^]_pm_ signaling in α- and β-cells

**Figure 6.**
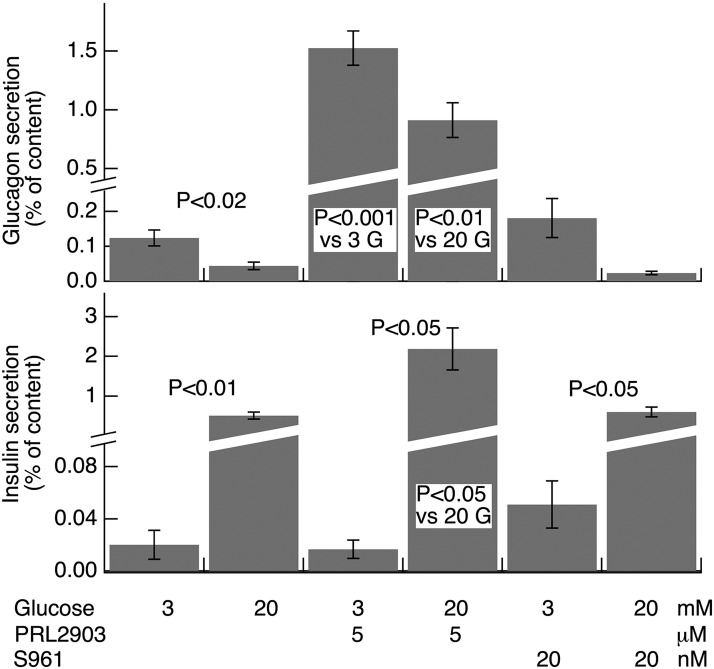
The SSTR-2 antagonist PRL2903 potently amplifies glucagon and insulin secretion. Glucagon and insulin secretion were measured after a 40 min incubation of mouse pancreatic islets in the presence of 3 or 20 mM glucose and a 20 nM concentration of the insulin receptor antagonist S961 or a 5 μM concentration of the SSTR-2 antagonist PRL2903. Secretion is expressed as a percentage of the total islet hormone content. Bar: means ± sem of results in 7 experiments. There were statistically significant differences between the experimental groups (ANOVA; *P* < 0.001). The *post hoc* significance levels for the effect of glucose elevation are indicated above the bars and those for the effect of PRL2903 within the bars.

In view of the unexpected synchronization between [Ca^2+^]_pm_ oscillations in α- and β-cells exposed to 20 mM glucose, we explored the possibility that glucagon secretion under these conditions is determined by paracrine factors independent of [Ca^2+^]_pm_. Both somatostatin and insulin have been implicated in glucose regulation of glucagon release (36, 37), and we therefore compared how receptor antagonists to these hormones affected glucagon and insulin release from islets in relation to their effects on [Ca^2+^]_pm_. An increase in glucose from 3 to 20 mM stimulated insulin release 25-fold and inhibited glucagon secretion by 65% (**Fig. 6**). The insulin receptor antagonist S961 did not significantly affect glucagon or insulin release at either glucose concentration, whereas the SSTR-2 antagonist PRL2903 potently stimulated glucagon release at both 3 (12-fold) and 20 (21-fold) mM glucose. However, in accordance with a previous observation (20), the presence of PRL2903 did not seem to prevent a 40% inhibition of glucagon release by 20 mM glucose, although this effect did not reach statistical significance in the present study. Whereas basal insulin secretion at 3 mM glucose was unaffected by PRL2903, secretion stimulated by 20 mM of the sugar was amplified 4-fold. The latter effect is probably indirectly mediated by the pronounced increase in glucagon release, in that SSTR-2 dominates rodent α-cells, whereas the β-cells essentially express type 5 receptors (38).

**Figure 7.**
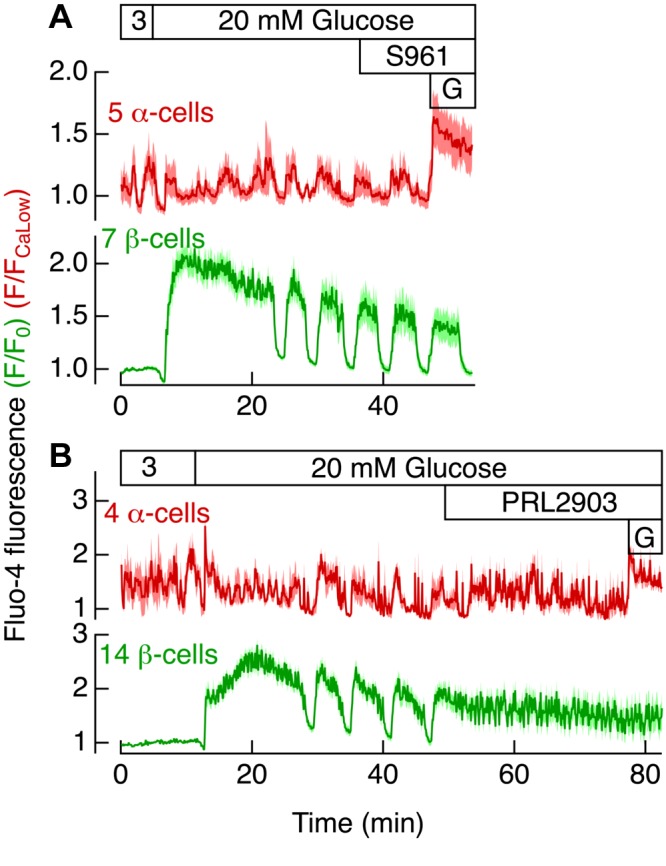
Insulin receptor and SSTR-2 antagonists have modest effects on [Ca^2+^]_pm_ oscillations in α- and β-cells. *A*) Average [Ca^2+^]_pm_ data for 5 α-cells (dark red) and 7 β-cells (dark green) ± sem (pink: α-cells; light green: β-cells) within the same islet. *B*) Corresponding average data ± sem from 4 α- and 14 β-cells. Fluo-4 fluorescence in α-cells was normalized to that corresponding to the lowest [Ca^2+^]_pm_ values (*F*/*F*_CaLow_) and in β-cells to initial [Ca^2+^]_pm_ (*F*/*F*_0_). *A*, *B*) The glucose concentration was increased from 3 to 20 mM, and a 20 nM concentration of the insulin receptor antagonist S961 (*A*) or a 5 μM concentration of the SSTR antagonist PRL2903 (*B*) and 1 mM glutamate (G) was added as indicated.

S961 had no effect on [Ca^2+^]_pm_ oscillations in 5 α- and 7 β-cells exposed to 20 mM glucose (**Fig. 7*A***), whereas PRL2903 transformed glucose-induced slow oscillations of average [Ca^2+^]_pm_ in 14 β-cells into faster oscillations from an elevated plateau (Fig. 7*B*), which is characteristic of a response mediated by glucagon (39). The PRL2903 effect on average [Ca^2+^]_pm_ in 4 α-cells was much more subtle, as shown by recordings from the same islet (Fig. 7*B*). If anything, a tendency toward a slow oscillatory pattern in 20 mM glucose became less obvious after addition of PRL2903. Similar observations were made in 17 α- and 20 β-cells in 3 islets from 2 mice (S961) and in 22 α- and 39 β-cells in 5 islets from 3 mice (PRL2903).

## DISCUSSION

The relatively low proportion of α-cells within pancreatic islets hampers study of the signaling mechanisms that mediate glucose inhibition of glucagon secretion. Attempts to circumvent this difficulty by purifying α-cells from rat (40) and mouse (15) islets with fluorescence-activated cell sorting led to the unexpected discovery that glucose stimulates glucagon secretion in this preparation. However, it is possible to study individual islet cells in their native location within pancreatic islets using confocal or TIRF imaging techniques. The latter approach has the advantage of accessing only the most superficial layer of islet cells, which is α-cell-rich in rodent islets (41). Cell identification by immunostaining is usually not feasible, because islet morphology is sufficiently perturbed to prevent confident association of specific responses to cell type. That α-cells show oscillatory Ca^2+^ activity at low glucose concentrations when Ca^2+^ in the β-cells is low and stable and that Ca^2+^ signaling in the β-cells is activated by glucose elevation have been used with confocal microscopy for cell identification within pancreatic islets (42). These criteria, together with the α-cell-characteristic glutamate response (35) and differences in cell size reflected by the footprint areas, were now used to discriminate between the 2 cell types. The use of transgenic GLU-RFP mice considerably simplified preliminary identification of the α cells, although immunostaining showed that it is not perfect. Therefore, we always complemented RFP-based α- and β-cell identification with functional cytoplasmic Ca^2+^ concentration ([Ca^2+^]_i_) characteristics. Indeed, because of the strong interference between Perceval and RFP, we relied entirely on the functional [Ca^2+^]_i_ responses for the ATP measurements. We were surprised that no Perceval-RFP interference was reported when seemingly identical GLU-RFP mice were used to record the α-cell ATP response to a 1–6 mM glucose increase with confocal microscopy (25). We found similar interference in control experiments with confocal microscopy. It cannot be attributed to fluorescence bleed-through or altered cell morphology and may involve changes in Ca^2+^ or ATP or both, in that K^+^ depolarization also induced parallel changes in RFP and Perceval fluorescence.

Although in prior studies, we calibrated the Perceval fluorescence signal in permeabilized β-cells exposed to different ATP concentrations (29, 43), it is difficult to perform such calibration in intact islets. Therefore, the present approach does not allow direct comparison of the ATP concentrations in α- and β-cells. In agreement with previous static ATP measurements on purified rat islet cell populations (23) and kinetic recordings on mouse islets with luciferase-expressing α-cells (11, 14), glucose elevation induced lesser ATP increases in α- than in β-cells, which is consistent with a lower rate of glucose oxidation in α-cells (23, 44) and lesser oxidative phosphorylation efficiency caused by high expression of uncoupling protein 2 (45). Static measurements of adenine nucleotides in α-cell-enriched rat islet fractions have indicated that the total ATP concentration is ∼2.2-fold higher than in β-cells at 1 mM glucose (23). Therefore, it is possible that [ATP]_pm_ is more elevated in α-than in β-cells exposed to low glucose concentrations. When expressed as a percentage of the subsequent response to 20 mM, glucose elevation from 1 to 5 mM induced a much stronger [ATP]_pm_ response in α- than in β-cells. The higher α-cell sensitivity at low glucose concentrations is consistent with ATP’s involvement in glucose control of glucagon secretion during recovery from hypoglycemia.

Glucose-stimulated insulin release is pulsatile (4, 5), and the pulses reflect coinciding elevations of the concentrations of triggering Ca^2+^ and amplifying cAMP, which show synchronized oscillations in β-cells (31, 46). Although an increase in ATP may affect both Ca^2+^ and cAMP, the interplay between the messengers is complex, because ATP consumption increases in response to Ca^2+^ elevation. The β-cells within islets are coupled by gap junctions (47), leading to effective synchronization of Ca^2+^ signaling (34, 42), which explains why average [ATP]_pm_ is related to average [Ca^2+^]_pm_ in the same manner as in single β-cells (29). Glucose stimulation thus resulted in an early ATP elevation that slightly preceded the initial increase in Ca^2+^, but the subsequent oscillations of ATP and Ca^2+^ were essentially antiparallel. Our analysis of α-cells indicated a similar inverse relationship between changes in the levels of [Ca^2+^]_pm_ and [ATP]_pm_. At 3 mM glucose, when the α-cells, in accordance with previous observations (42), did not show apparent synchronization of the [Ca^2+^]_pm_ oscillations, the inverse relation to [ATP]_pm_ was evident only in individual α-cells. However, at 20 mM glucose, the [Ca^2+^]_pm_ oscillations tended to synchronize among the α-cells. This synchronization was not as unmistakable as between the electrically coupled β-cells but became distinct after [Ca^2+^]_pm_ of several α-cells within an islet was averaged, probably explaining why it has previously escaped detection (42). In this situation, the phase shift between the average [Ca^2+^]_pm_ and [ATP]_pm_ oscillations in the α cells seemed identical to that in the β-cells and probably reflects energy-dependent Ca^2+^ extrusion.

At 20 mM glucose, glucagon release from isolated islets also is pulsatile and in opposite phase to the insulin pulses (4, 5), which likely underlies the same phase relationship of the hormone oscillations in the circulation (1, 2). Ca^2+^ is the major trigger of insulin secretion (8) and is generally believed to underlie stimulation of glucagon release (16–21). As a consequence, one would expect that the [Ca^2+^]_pm_ oscillations would be in opposite phase in α- and β-cells. We were therefore perplexed that the highly coherent [Ca^2+^]_pm_ oscillations in β-cells synchronized with the average [Ca^2+^]_pm_ oscillations in α-cells. This synchronization was consistent in islets from several preparations studied with different Ca^2+^ indicators and probably involves paracrine coordinating factors. In a previous study, it was noticed that although Ca^2+^ oscillations are not synchronized among α-cells, oscillations in individual α-cells exposed to 8–12 mM glucose sometimes approximately follow the surrounding coordinated β-cell waves of Ca^2+^ (15). Our data indicate that α- and β-cell Ca^2+^ synchrony dominates at glucose concentrations that stimulate insulin secretion. Therefore, glucose may act downstream of Ca^2+^, which is consistent with proposed Ca^2+^-independent inhibition by hyperglycemic concentrations of the sugar (48).

The present data support a recent suggestion that glucose inhibition of glucagon release is regulated by α-cell-intrinsic mechanisms during recovery from hypoglycemia and that paracrine factors become important when the glucose concentration exceeds the thresholds for stimulation of δ- and β-cell secretion (49). The observation that ATP in α-cells is relatively more sensitive to low glucose concentrations in α- than in β-cells is consistent with models that attribute a messenger role to ATP in glucagon secretion. Moreover, SSTR-2 antagonism potently stimulated glucagon release with little effect on [Ca^2+^]_pm_, as is expected if somatostatin acts downstream of Ca^2+^ (50, 51). Pulsatile secretion of glucagon in opposite phase to that of insulin and somatostatin (2, 4, 5) as a result may be generated at high glucose, although [Ca^2+^]_pm_ oscillations were found to be in the same phase in α- and β-cells. However, somatostatin cannot explain all inhibition; present and previous (20) data show that glucose also inhibits glucagon secretion after SSTR-2 blockade and when SSTR signaling is prevented (52, 53). No evidence was found for an inhibitory role of insulin but it remains to be clarified whether the somatostatin-independent inhibition involves other β-cell factors or reflects only an intrinsic α-cell mechanism.

## Supplementary Material

Supplemental Data

## Abbreviations

[ATP]_pm_: subplasma membrane concentration of ATP
[Ca^2+^]_i_: cytoplasmic Ca^2+^ concentration; [Ca^2+^]_pm_, subplasma membrane concentration of Ca^2+^
FBS: fetal bovine serum
GLU-RFP mice: transgenic mice expressing red fluorescent protein under proglucagon promoter control
K_ATP_ channels: ATP-sensitive K^+^ channels
RFP: red fluorescent protein
SSTR: somatostatin receptor
TIRF: total internal reflection fluorescence
