# Supplementary material for: Submembrane ATP and Ca2+ kinetics in α-cells: unexpected signaling for glucagon secretion
Source: FASEB J. 2015 Apr 24;29(8):3379–88. doi: 10.1096/fj.14-265918 (PMC4539996; doi:10.1096/fj.14-265918)
Supplement: Supplemental Data [file supp_29_8_3379__index.html]

Submembrane ATP and Ca2+ kinetics in α-cells: unexpected signaling for glucagon secretion — Supplemental Data 

# Submembrane ATP and Ca2+ kinetics in α-cells: unexpected signaling for glucagon secretion

## Supplemental Data

**Files in this Data Supplement:**

- Supplemental Data
- Supplemental Data
- Supplemental Data
- Supplemental Data
