# Supplementary material for: Submembrane ATP and Ca2+ kinetics in α-cells: unexpected signaling for glucagon secretion
Source: FASEB J. 2015 Apr 24;29(8):3379–88. doi: 10.1096/fj.14-265918 (PMC4539996; doi:10.1096/fj.14-265918)
Supplement: Supplemental Data [file supp_fj.14-265918_Supplemental_Figure1.docx]

**
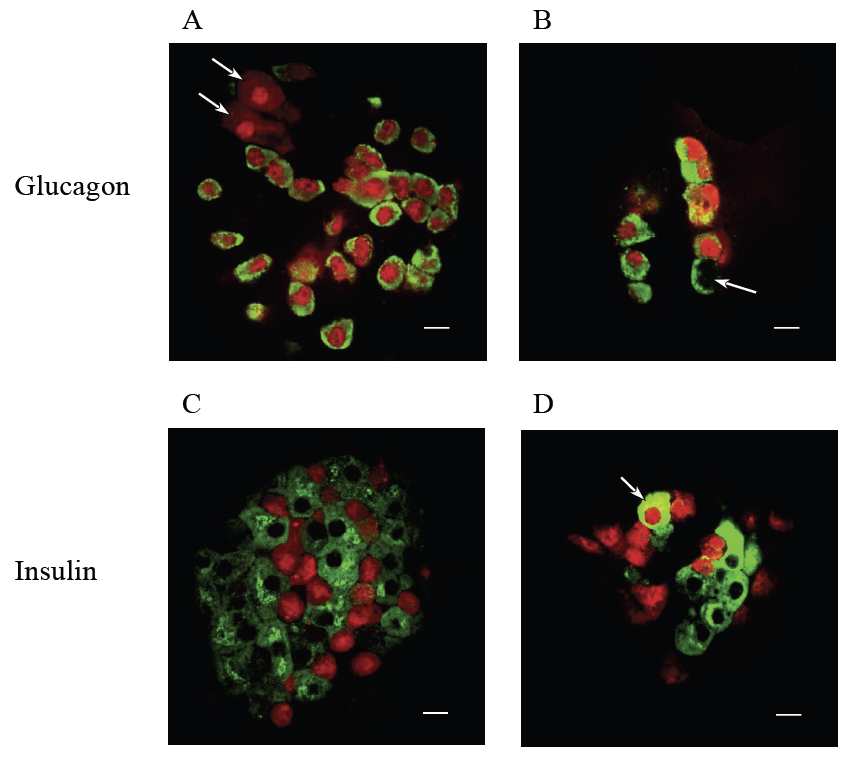
**

**Supplemental Figure S1.** Most RFP-expressing islets cells from GLU-RFP mice are glucagon positive and insulin negative. (A, B) Confocal images of glucagon immunostained islets exemplifying that 651 of 733 RFP expressing cells (89%) in 35 islets from 4 mice also expressed cytoplasmic glucagon. The arrows in (A) indicate two of RFP-expressing cells that are glucagon negative. The arrow in (B) shows a glucagon positive cell that does not express RFP (total 22 cells, 3%). (C) When 29 islets from the same 4 mice were immunostained for insulin there was no overlap between RFP and insulin and the RFP-expressing cells were generally smaller. (D) However, 63 of 467 cells with nuclear RFP-expression (13%) also showed cytoplasmic staining for insulin (arrow). Scale bars, 10 µm.
