# Supplementary material for: Submembrane ATP and Ca2+ kinetics in α-cells: unexpected signaling for glucagon secretion
Source: FASEB J. 2015 Apr 24;29(8):3379–88. doi: 10.1096/fj.14-265918 (PMC4539996; doi:10.1096/fj.14-265918)
Supplement: Supplemental Data [file supp_fj.14-265918_Supplemental_Figure2.docx]

**
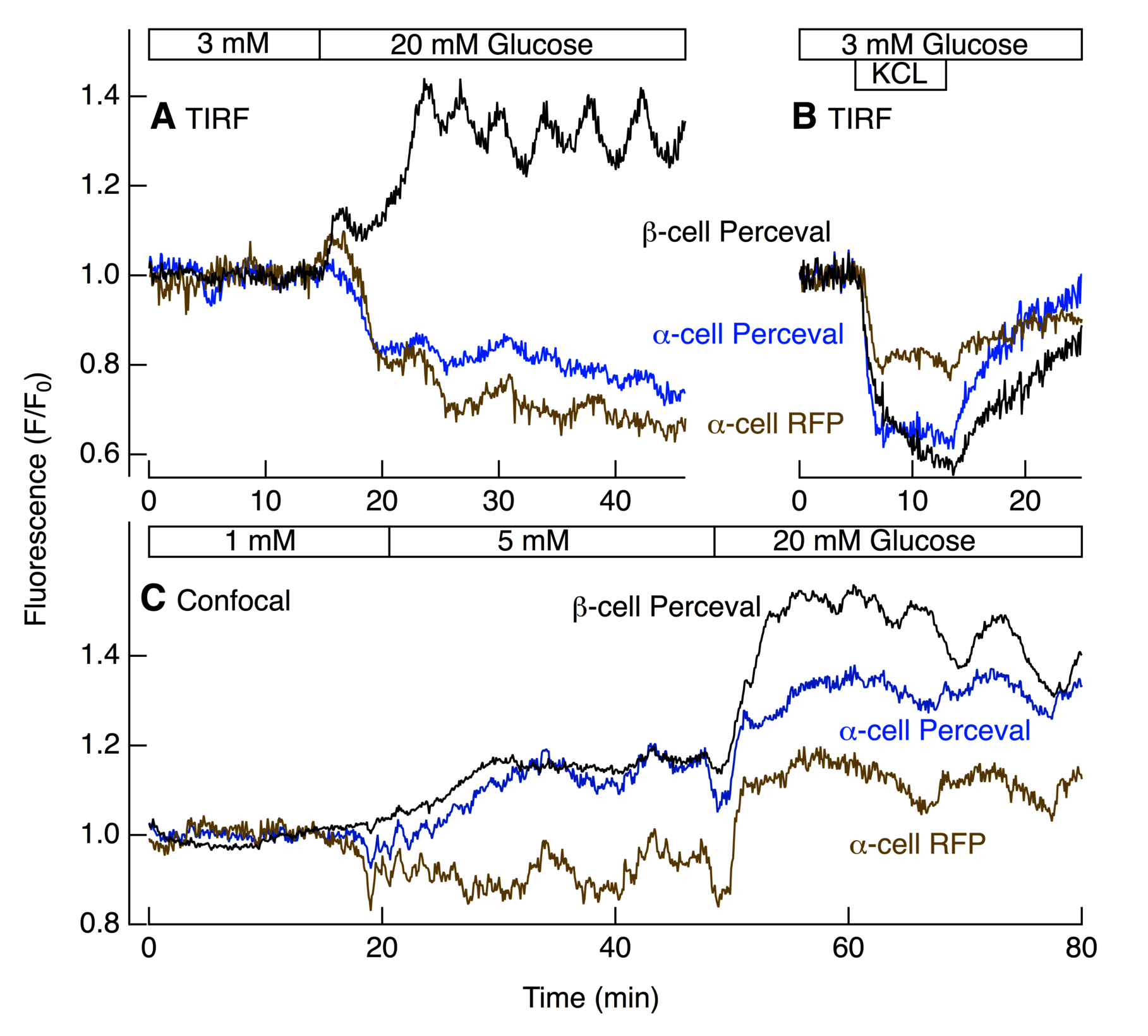
**

**Supplemental Figure S2.** Perceval reports [ATP]_pm_ in β‑cells but interferes with RFP in α‑cells from GLU-RFP mice. TIRF and confocal microscopy were used to record [ATP]_pm_ (Perceval fluorescence) and RFP fluorescence in individual cells within pancreatic islets from GLU-RFP mice. (A) The RFP-negative β‑cells responded to 20 mM glucose with rapid elevation of Perceval fluorescence that was temporarily interrupted followed by further increase and distinct slow oscillations, a pattern identical to our previous TIRF recordings in β‑cells from regular C57Bl/6J mice ([29](#_ENREF_29)). (A, B) However, although there was no spectral overlap between RFP and Perceval in the α‑cells, the fluorescence from the proteins showed erratic responses to increasing concentrations of glucose or K^+^ depolarization, often with co-variation in fluorescence. (C) Interference was also observed in confocal recordings on individual α‑cells within pancreatic islets when the glucose concentration was raised from 1 to 5 and 20 mM. The interference probably reflects molecular interactions between RFP and Perceval, since the RFP fluorescence was stable in α-cell recordings with a fluorescent Ca^2+^ indicator in the absence of Perceval (**Fig. 1**). Fluorescence (F) is normalized as the F/F_0_ ratio where F_0_ is the initial fluorescence.
