# Supplementary material for: Submembrane ATP and Ca2+ kinetics in α-cells: unexpected signaling for glucagon secretion
Source: FASEB J. 2015 Apr 24;29(8):3379–88. doi: 10.1096/fj.14-265918 (PMC4539996; doi:10.1096/fj.14-265918)
Supplement: Supplemental Data [file supp_fj.14-265918_Supplemental_Figure3.docx]

**
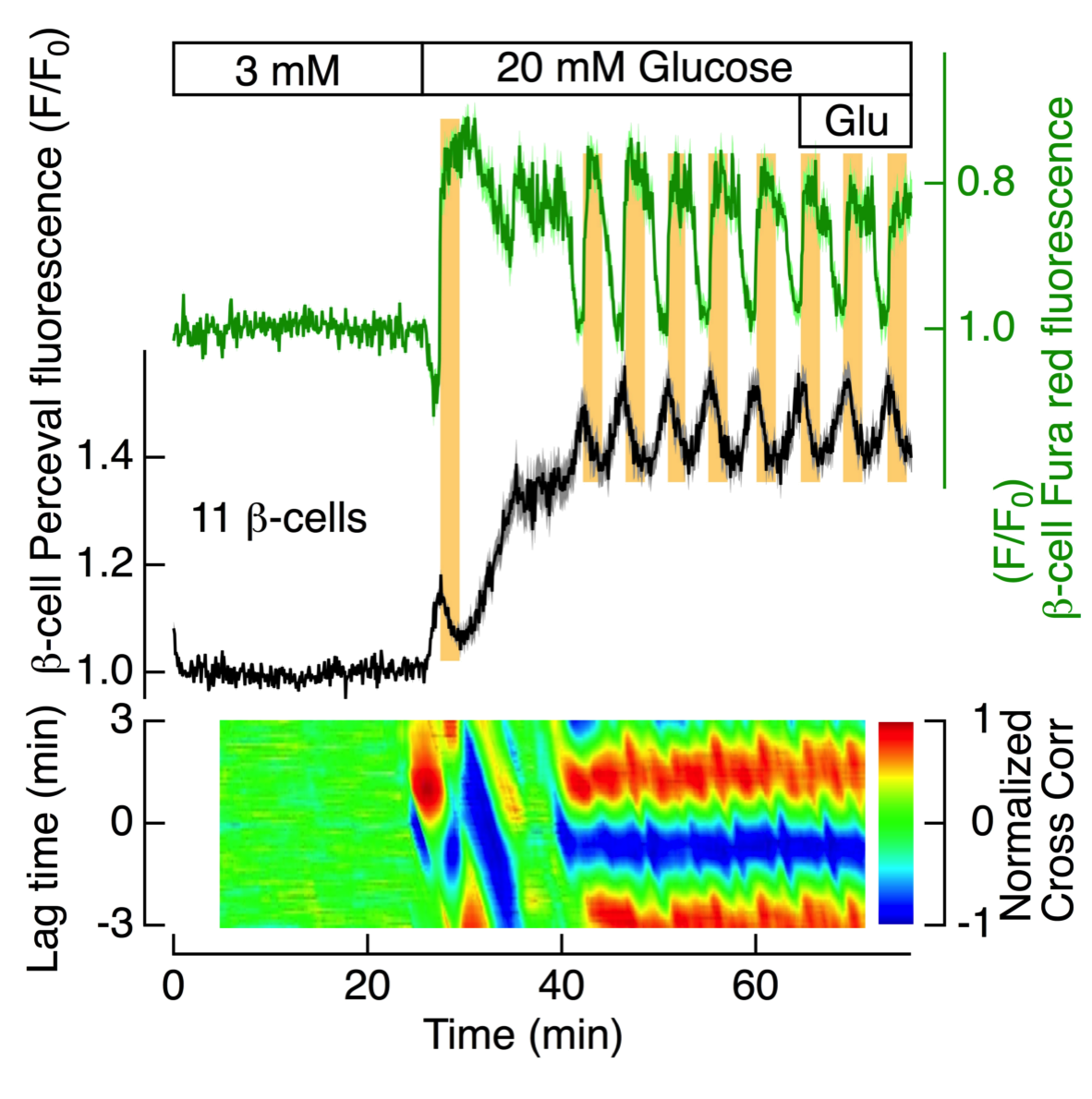
**

**Supplemental Figure S3** Glucose elevation raises β‑cell [ATP]_pm_ and induces oscillations in opposite phase to those of [Ca^2+^]_pm_**.** TIRF microscopy was used to record Fura Red and Perceval fluorescence in individual cells within a pancreatic islet from a normal mouse. Perceval and Fura Red fluorescence (F) are normalized as the F/F_0_ ratio where F_0_ is the initial fluorescence in 3 mM glucose, and the Fura Red scale is inverted to show increases of [Ca^2+^]_pm_ as positive deflections. The graphs show average data (dark green for [Ca^2+^]_pm_ and black for [ATP]_pm_) ± SEM (light green for [Ca^2+^]_pm_ and gray for [ATP]_pm_) for 11 β‑cells within the same islet. The islet was initially exposed to 3 mM glucose and the concentration was then increased to 20 mM as indicated. At the end of the experiment 1 mM glutamate (Glu) was added. The vertical yellow background areas are aligned to increases of the [Ca^2+^]_pm_ that correspond to lowerings of [ATP]_pm_. Correlation was calculated from consecutive pairs of data segments of 4 min duration and shifted 5 s in time in relation to the previous segment. The two-dimensional cross correlogram (major colored area) was constructed as described in the legend to Fig. 3. The data were obtained from the same islet studied in Figures 3-4 and S4.
