# Supplementary material for: Submembrane ATP and Ca2+ kinetics in α-cells: unexpected signaling for glucagon secretion
Source: FASEB J. 2015 Apr 24;29(8):3379–88. doi: 10.1096/fj.14-265918 (PMC4539996; doi:10.1096/fj.14-265918)
Supplement: Supplemental Data [file supp_fj.14-265918_Supplemental_Figure4.docx]

**
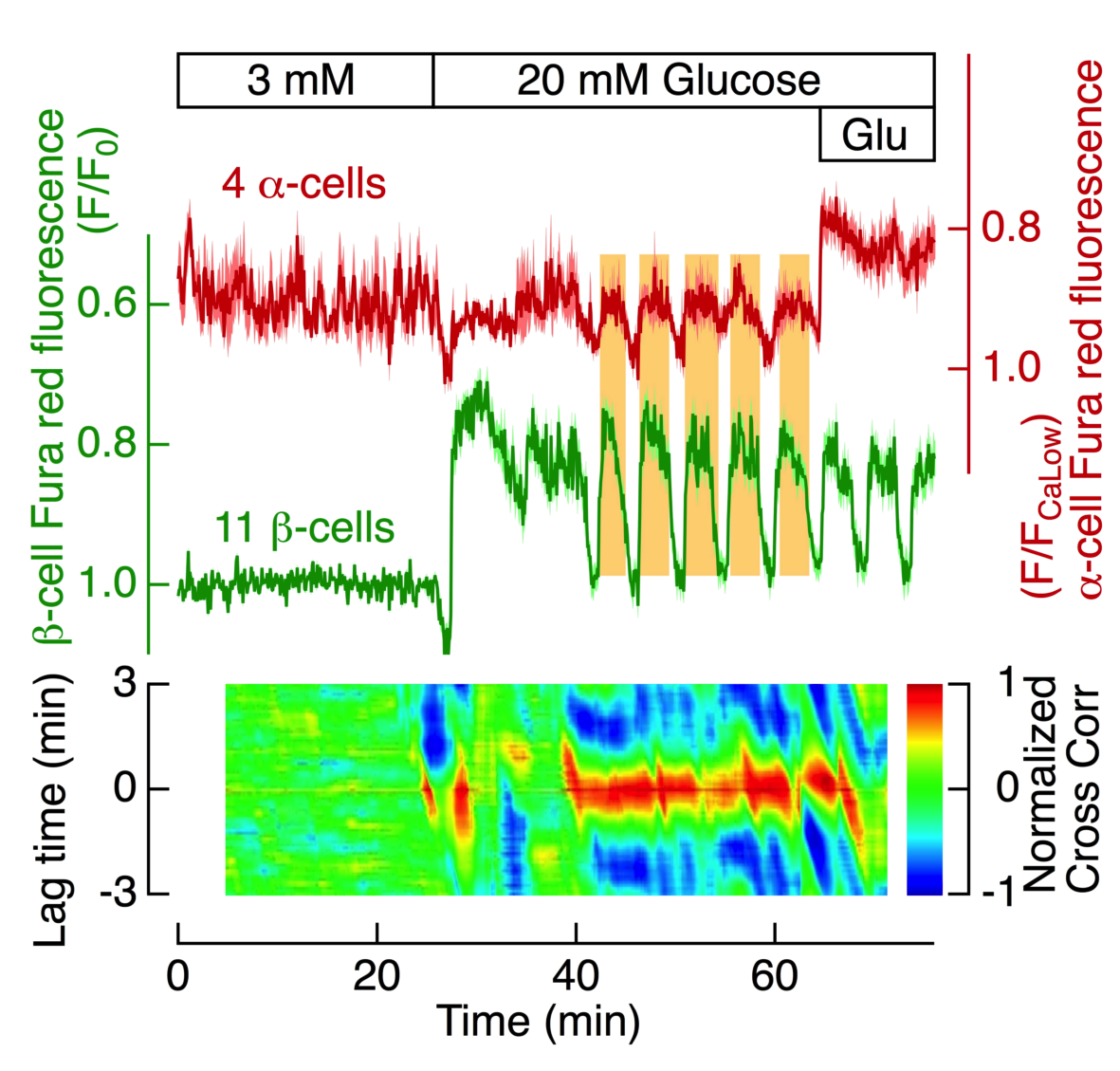
**

**Supplemental Figure S4.** Glucose-induced [Ca^2+^]_pm_ oscillations are synchronized between α‑cells and β‑cells. The graphs show average [Ca^2+^]_pm_ data (red for α‑ and dark green for β‑cells) ± SEM (pink for α‑ and light green for β‑cells) for 4 α‑cells and 11 β‑cells within same islet. Fura Red fluorescence F in α‑cells is normalized to that corresponding to the lowest [Ca^2+^]_pm_ values (F/F_CaLow_) and in β‑cells to initial [Ca^2+^]_pm_ (F/F_0_). The scale is inverted to show increases of [Ca^2+^]_pm_ as positive deflections. The glucose concentration was increased from 3 to 20 mM as indicated and 1 mM Glutamate (Glu) was added at the end of the experiment. The vertical yellow background areas are aligned to glucose-induced peaks of the [Ca^2+^]_pm_ oscillations in the β‑cells. Correlation was calculated from consecutive pairs of data segments of 4 min duration and shifted 5 s in time in relation to the previous segment. A two-dimensional cross correlogram (major colored area) was constructed from consecutive one-dimensional cross correlations with time on the X axis and the lag time of the correlation on the Y axis, and the normalized cross correlation amplitude coded in color (vertical color bar). The data were obtained from the same islet studied in Figures 3-4 and S3.
